# Niclosamide inhibits ovarian carcinoma growth by interrupting cellular bioenergetics

**DOI:** 10.7150/jca.41418

**Published:** 2020-03-13

**Authors:** Fugen Shangguan, Yan Liu, Li Ma, Guiwu Qu, Qing Lv, Jing An, Shude Yang, Bin Lu, Qizhi Cao

**Affiliations:** 1 Protein Quality Control and Diseases Laboratory, Attardi Institute of Mitochondrial Biomedicine, School of Laboratory Medicine and Life Sciences, Wenzhou Medical University, Wenzhou, Zhejiang 325035, P.R. China; 2 Key Laboratory of Diagnosis and Treatment of Severe Hepato-Pancreatic Diseases of Zhejiang Province, the First Affiliated Hospital of Wenzhou Medical University, Wenzhou, Zhejiang, 325000, China.; 3 Department of Immunology, School of Basic Medical Sciences, Binzhou Medical University, Yantai, Shandong 264003, P.R. China; 4 Anti-aging Research Institution, Binzhou Medical University, Yantai, Shandong 264003, P.R.China; 5 School of Medicine, University of California - San Diego, La Jolla, CA 92037, USA; 6 School of Agriculture, Ludong University, Yantai, Shandong 264025, P.R.China

**Keywords:** Niclosamide, ovarian carcinoma, cellular bioenergetics, MEK1/2-ERK1/2 signal

## Abstract

**Background:** Ovarian carcinoma is a common malignant tumor of the female reproductive organs with an incidence rate second only to cervical and endometrial cancers. In the past 10 years, anticancer therapy has focused on Niclosamide, an anthelmintic teniacide that is commonly used against tapeworms and has been approved for use in humans for nearly 50 years. Importantly, Niclosamide has been confirmed to target the Wnt/β-catenin, mTOR, STAT3, NF-κB, and Notch pathways has been widely investigated in multiple cancer types. However, the potential benefits of Niclosamide therapy for treatment of ovarian carcinoma have not been established.

**Methods:** CCK-8 colony formation assays were performed to evaluate cell viability and tumor growth. Cell apoptosis was measured by flow cytometry. A Seahorse XF96 analyzer was used to measure cellular bioenergetics. Mito-tracker stained mitochondria were visualized by confocal microscopy. Western blotting was used to detect expressed proteins. A nude mouse transplanted-tumor model was used to evaluate the antitumor activity of Niclosamide in ovarian carcinoma.

**Result:** Niclosamide treatment significantly suppressed ovarian carcinoma growth and induced cell apoptosis by inactivating MEK1/2-ERK1/2 mediated signal transduction. Overall, mitochondrial respiration and aerobic glycolysis were both decreased by Niclosamide treatment. Niclosamide dramatically enhanced ROS-activated and JNK-mediated apoptosis in cells subjected to glucose deprivation. Niclosamide also showed *in vivo* antitumor activity in the nude mouse transplanted-tumor model.

**Conclusion:** Collectively, these data highlight a novel anti-tumor mechanism of Niclosamide that involves an interruption of cell metabolism. The finding also indicates a potential for the application of Niclosamide in ovarian carcinoma therapy.

## Introduction

Ovarian carcinoma is a common malignant tumor of the female reproductive organs with an incidence rate second only to cervical and endometrial cancers 1. The most common type of ovarian cancer is epithelial cancer, followed by malignant germ cell tumors. The mortality of ovarian epithelial cancer ranks first among various gynecological tumors and poses a serious threat to women's lives. The poor prognosis of this disease is due to difficult in an early diagnosis because of the anatomical location of the ovaries deep in the pelvic cavity, the small size of the ovaries, and the lack of typical symptoms of this cancer. In patients with ovarian epithelial cancer, less than 30% of the tumors are confined to the ovary, and most of tumors spread to the pelvic and abdominal organs, thereby further complicating early diagnosis.

In recent years, the survival of patients with ovarian cancer has been prolonged by the adoption of targeted therapy for poly ADP-ribose polymerase (PARP) vascular endothelial growth factor receptor (VEGFR) 2, 3. Immunotherapeutic approaches are primarily aimed at the use of immunological checkpoint inhibitors, including PD-1/PD-L1 inhibitors and inhibitors of CTLA-4 4. However, the effective rate of a single drug is still only about 10%, so the efficacy of combined drugs needs further study. In particular, side effects and drug resistance during treatment remain important issues requiring greater consideration. Drugs with few side effects and high safety are clearly needed.

Niclosamide (amuricide or molluscicide) is an anthelmintic salicylamide derivative that inhibits mitochondrial oxidative phosphorylation in the cells of parasitic worms, thereby restricting ATP production. This causes the worms to detach from the intestinal wall and be excreted with the feces 5. Mitochondria are the major source of ATP production in mammalian cells, however, in tumor cells, glycolysis is commonly hyper-activated which indicates decreased activity of mitochondrial function. Dr. Otto Warburg found tumor cells dependent on glycolysis to support cell growth even under normoxic condition which named “Warburg effect”. Cell metabolic reprogramming is one of the hallmarks of tumor cells and targeting cell metabolism is considered as a potential strategy for cancer therapy. In recent years, Niclosamide has been widely investigated in cancer research and has shown effective inhibition of multiple cancer-associated signal pathways. High-throughput screening data showed that Niclosamide is an inhibitor of Wnt/β-catenin signaling and disrupts β-catenin/TCF complex formation while promoting Wnt co-receptor LRP6 degradation 6-9. Several studies have evaluated the anti-tumor activity of Niclosamide in suppressing prostate cancer, breast cancer, osteosarcoma, and colorectal carcinoma. Niclosamide has no significant inhibitory effect on mTORC2, indicating that catalytic activity of mTOR is not prohibited by Niclosamide but the signaling to mTORC1 is suppressed. Mechanistically, the mTORC1 signal suppression by Niclosamide depends on inhibition of the lysosomal degradative function 10, 11. JAK-STAT signaling also appears to be a potential target of Niclosamide in multiple myeloma and human lung cancer cells 12, 13. Similarly, repression of NF-κB and Notch signaling by Niclosamide has also been demonstrated. In acute myelogenous leukemia and primary human glioblastoma cells, Niclosamide suppresses TAK1→IKK→IκBα→NF-κB signal transduction cascades and the expression of cleaved activated Notch1 receptor 14, 15. Recently, Niclosamide was also shown to enhance the efficacy of PD-1/PD-L1 immune checkpoint blockade and the cytotoxicity of cisplatin in non-small cell lung cancer (NSCLC) cells 16. Multiple novel Niclosamide analogs have also been developed and confirmed to have potent anti-tumor activity with reduced biological toxicity 17.

Recently, Niclosamide and its analogs have also been verified to suppress ovarian carcinoma cell growth by targeting the Wnt/β-catenin, JAK2/ STAT3, and mTOR signal pathways, as previously described for other cancer types 17. However, an unanswered question is whether Niclosamide has a role in the regulation of other signal pathways with known involvement in cancer progression. Therefore, in the present study, we investigated the possible role of Niclosamide in regulating cellular bioenergetics, and we identified a potential mechanism for the inhibition of ovarian carcinoma by Niclosamide. Our data identify Niclosamide as effective suppressor of ovarian carcinoma growth, and our findings highlight a novel disruption in cell metabolism that can explain the anti-tumor activity of Niclosamide.

## Materials and Methods

### Reagents and antibodies

Horseradish peroxidase (HRP)-conjugated anti-rabbit, anti-mouse immunoglobulin G, bradford protein assay kit, and cell counting kit-8 (CCK-8 kit) were obtained from Beyotime (Shanghai, China). Annexin V-fluorescein isothiocyanate (FITC)/ propidium iodide (PI) apoptosis detection kit was purchased from BD (San Jose, CA, USA). Giemsa and crystal violet were purchased from Solarbio Bioscience & Technology (Shanghai, China). Trypan blue was obtained from Life Technologies (Carlsbad, CA). BCA protein assay kit and Pierce ECL western blotting substrate were obtained from Beyotime Biotechnology (Shanghai, China). Intact cellular oxygen consumption rate (OCR) and extracellular acidification rate (ECAR) assay kits were purchased from Seahorse Bioscience Company (North Billerica, MA). Glucose was obtained from Sigma (St. Louis, MO). Protease (Complete Mini) and phosphatase (PhosphoSTOP) inhibitor cocktail tablets were purchased from Roche Applied Science (Indianapolis, IN). DAPI (C1002) and NAC (616-91-1) were purchased from Beyotime (Shanghai, China). Mitotracker (#9082) was purchased from Cell Signaling Technology (Beverly, MA). Monoclonal antibody against Actin was obtained from Abclonal (WuHan, China). Antibodies recognizing p-MSK1 (#9595), p-MEK1/2 (#9154), MEK1/2 (#4694), p-ERK1/2 (#4370), PKM2 (#4053), p-JNK (#4668), JNK (#9525) were obtained from Cell Signaling Technology (Beverly, MA). Antibodies against ERK1/2 (67170-1-Ig), K-RAS (12063-1-Ig) and OMA1 (17116-1-Ig) were purchased from Proteintech (Wuhan, China). The primary antibodies against HK2 (500994), PFKM (505477), PGK1 (501965), and LDHA (501146) were obtained from Zen Bioscience (Chengdu, China). The primary antibodies recognizing OPA1 (612606) was purchased from BD-Pharmingen (San Jose, CA, USA).

### Cell lines and cell culture

The SKOV3, HO8910 human ovarian cancer cell lines were obtained from the Cell Bank of the Chinese Academy of Sciences (Shanghai, China). SKOV3 cells were cultured in RPMI-1640 medium (Life Technologies, Grand Island, NY) supplemented with 10% fetal bovine serum (FBS, Gemini, USA) and antibiotics (100 units/mL penicillin and 100 units/mL streptomycin) and HO8910 cells were cultured in DMEM medium supplemented with 10% fetal bovine serum and antibiotics. The cells were incubated at 37°C in a humidified incubator with 5% CO_2_. When the cultures reach approximately 50-70% confluence, the cells were treated with various concentrations of drugs. Dimethyl sulfoxide (DMSO) was used as a vehicle control. All cell lines were mycoplasma free and authenticated by the Cell Bank of the Chinese Academy of Sciences.

### Cytotoxic and anti-proliferation assays

To determine the inhibitory concentration (IC_50_) values of compounds, SKOV3 and HO8910 cells were seeded in 96-well plates at a density of 1×10^4^ cells/well and incubated overnight at 37 °C with 5% CO_2_. Cells were then treated with different concentrations (0, 0.5, 1, 2, 4, 8, 16, or 32 μM) of Niclosamide or DMSO (vehicle control) for 48 hr, followed by Niclosamide incubating with CCK-8 for another 3 hr at 37°C. The absorbance at 450 nm was measured using a Varioskan Flash microplate reader (Thermo Scientific, Waltham, MA). Each assay was performed in triplicate and data was derived from at least three independent experiments.

The effects of Niclosamide on the proliferation of SKOV3 and HO8910 cell lines were evaluated using Cell Counting Kit-8 (CCK-8). Briefly, SKOV3 andHO8910 cells were seeded into 96-well plates at a density of 3×10^3^ cells/well and incubated overnight at 37°C with 5% CO_2_. Cells were then treated with gradient concentrations (0, 4, and 8 μM) of Niclosamide for 1, 2, 3 and 4 days, and followed by incubating with Cell Counting Kit-8 (CCK-8) for 3 hr at 37°C, respectively. The absorbance at 450 nm was measured using a Varioskan Flash microplate reader (Thermo Scientific, Waltham, MA).

### Colony formation assay

Both SKOV3 and HO8910 cells were counted and a total of 800 cells per well were seeded evenly into 6-well plates and incubated at 37°C for 7-10 days in a humidified incubator with 5% CO_2_. After treatment with gradient concentrations (0, 4, and 8 μM) of Niclosamide for another 2-4 days or with Niclosamide without glucose for another 12 hr, cells were washed with pre-warmed PBS three times, fixed with 4% PFA, and stained with Giemsa solution for 15 min. Colonies were counted by two independent investigators.

### Western blot analysis

The ovarian cancer samples were washed three times with ice-cold PBS and homogenized using a homogenizer (Kinematica AG, Luzern, Switzerland) in 1.5 mL tissue RIPA lysis buffer (50 mM Tris-HCl, pH 7.4, 1.0% Triton X-100, 1% sodium deoxycholate, 0.1% SDS and 150 mM NaCl) supplemented with protease inhibitor cocktail tablet, NaF (1 mM) and Na_3_VO_4_ (1 mM). Tissue homogenates were cleared by centrifugation at 13,000 rpm for 25 min at 4°C, and the supernatants were collected in clean microcentrifuge tubes on ice. A similar procedure was used to prepare whole cell extracts from cells. Briefly, SKOV3 and HO8910 cells were washed with ice-cold PBS and lysed in RIPA lysis buffer supplemented with protease and phosphatase inhibitors on ice for 20 min, followed by centrifugation at 13,000 rpm for 30 min at 4°C, and the supernatants were collected. Protein concentrations of the tissue homogenates or whole cell extracts were determined using the Pierce BCA protein assay kit. Tissue or cell extracts equivalent to 20 μg total protein were resolved in 10% SDS-PAGE gels followed by electrophoretic transfer onto PVDF membrane (0.22 μM, Bio-Rad, Hercules, CA) in Tris-glycine buffer. Blots were blocked at room temperature for 1.5 hr in 5% non-fat milk in Tris-buffered saline (TBS)-Tween (TBS-T) on a shaker, and then incubated with the primary antibodies in 5% non-fat milk TBS-T overnight at 4°C. The membrane was washed in TBS-T for at least 3 × 10 min and then incubated with horseradish peroxidase (HRP)- conjugated anti-rabbit or anti-mouse immunoglobulin G at room temperature for 1 hr with gentle shaking. Immunoreactive proteins were detected by ECL reagent according to the manufacturer's protocol (Biyetime bioteconology).

### FACS analysis for cell apoptosis

For apoptosis analysis, the SKOV3 and HO8910 cells were treated with gradient concentrations of Niclosamide (0, 4, and 8 μM) for 24 hr or with Niclosamide and/or NAC (10mM) in the presence or absence of glucose for 12 hr; cells were then collected and incubated with Annexin V-FITC/ PI (BD, San Jose, CA) in the dark at room temperature for 20 min, according to the manufacturer's protocol. Thereafter, cell samples were analyzed immediately using a BD AccuriTM C6 flow cytometer (BD, Franklin Lakes, NJ).

### XF Extracellular Flux Analyzer Experiments

The intact cellular oxygen consumption rate (OCR) and the extracellular acidification rate (ECAR) in compound treated SKOV3 and HO8910 cells were measured using a Seahorse XF-96 Extracellular Flux Analyzer (Seahorse Bioscience, North Billerica, MA) as described previously. Briefly, 80 μL Single-cell suspensions of SKOV3 and HO8910 cells were plated in XF96 cell culture microplates (Seahorse Bioscience) at a cellular density of 30,000 cells. The next day, both SKOV3 and HO8910 cells were treated with gradient concentrations of Niclosamide (0, 4, and 8 μM) for 8 hr. For OCR determinations, cells were incubated in base assay medium (according to manufacturer's instructions) supplemented with 2 mM glutamine, 10 mM glucose, and 1 mM pyruvate for 1 hr, prior to the measurements using the XF Cell Mito Stress Kit (Seahorse Bioscience). The final concentrations of Oligomycin, FCCP, and rotenone were 0.1 μM. For glycolytic metabolism measurements, cells were incubated in basal media prior to injections using the Glycolytic Test kit (Seahorse Bioscience). Results were obtained from three independent experiments; each with 8 replicates of each group of cells. At the end of each assay, a BCA protein assay kit was used to determine and normalize the protein concentrations, according to the manufacturer's instructions.

### Fluorescence detection in cultured cells

SKOV3 and HO8910 cells were treated with gradient concentrations of Niclosamide (0, 4, and 8 μM) for 24 hr and then stained with 50 nM Mitotracker red for 30 mins at 37 °C and fixed subsequently with 4% polyformaldehyde for 15 min. After that, cells were incubated with DAPI for 5 mins and washed three times with PBS. Finally, slides were examined with White Light Laser Confocal Microscope Leica TCS SP8 X (Leica) and the fluorescence images were collected using a Delta Vision OMX 3D-strucured illumi-nation microscope.

### Xenograft tumor assay

Female athymic nude mice were purchased from Shanghai Laboratory Animal Center, CAS (Shanghai, China) and housed in a specific pathogen-free (SPF) environment. For *in vivo* tumorigenesis analysis, nude mice at the age of 5 weeks were injected subcutaneously in the left flanks with 5 x 10^6^ of SKOV3 cells in 0.1 mL serum-free PBS. When the tumor volume has reached approximately 200 mm^3^, the mice were randomly sorted into two groups (n = 6/each group). Niclosamide suspension (20 mg/kg) was injected via intraperitoneal perfusion, once a day, for two consecutive weeks. At the same time, the control group was injected with the same volume of castor oil. The percentages of growth inhibition were defined as the ratio of tumor weight to that in the vehicle control. Tumor dimensions were determined using calipers, and the tumor volume (mm^3^) was calculated using the following the formula: volume = length × (width)^ 2^/2. The mice were sacrificed and the tumors were harvested and weighted. All animal studies were performed with a protocol approved by the Institutional Animal Care and Use Committee of Wenzhou Medical University.

### Statistical analysis

All statistical analyses were performed with the SPSS 16.0 statistical software package (SPSS Standard version 16.0, SPSS Inc., Chicago, IL). Data are shown as the mean ± SD from at least three independent experiments. Groups of 2 were analyzed with two-tailed students t test, groups greater than 2 with a single variable were compared using one-way ANOVA analysis with Tukey post hoc test, p < 0.05 was considered statistically significant.

## Results

### Potent anti-tumor activity of Niclosamide in ovarian carcinoma

**Figure 1:**
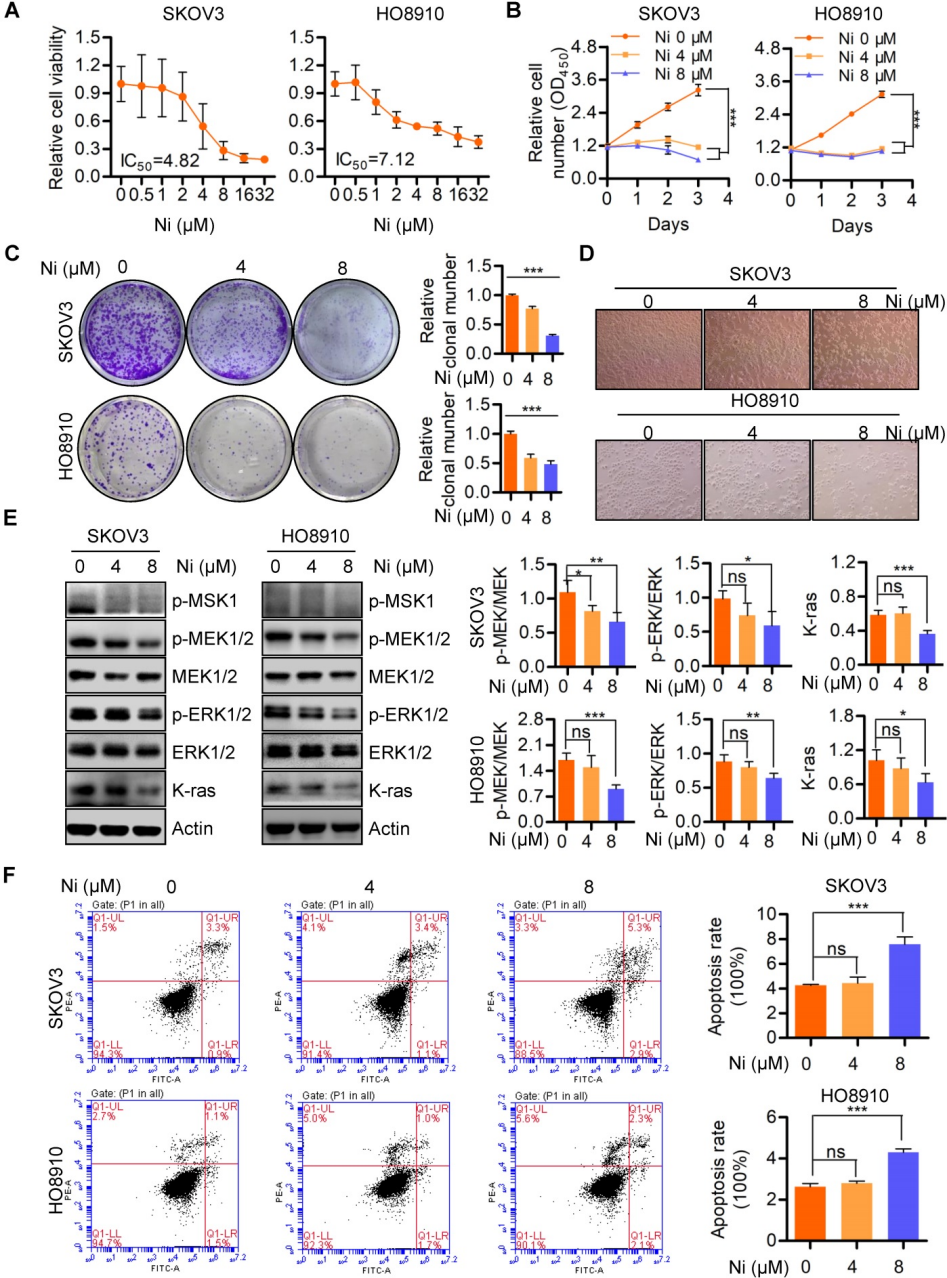
** Niclosamide effectively suppresses ovarian carcinoma cell growth. A and B.** Both SKOV3 and HO8910 ovarian cancer cell lines were treated with a gradient concentration of Niclosamide for 48 hr (**A**) and 96 hr (**B**), respectively. The cell viability was determined by either a CCK-8 assay (**A**) or a CCK-8 Cell Proliferation and Cytotoxicity Assay Kit (**B**) according to the manufacturer's instructions. **C.** SKOV3 and HO8910 cells were treated with different concentrations of Niclosamide and cultured for 3 days. Representative images of colonies as well as total colonies were recorded and measured. The data presented in right graphs represent the mean ± SD. **D.** Representative morphological changes of SKOV3 and HO8910 cells in response to different concentrations of Niclosamide. **E.** Western blotting analyses of p-MSK1, p-MEK1/2, MEK1/2, p-ERK1/2, ERK1/2 and K-ras in SKOV3 and HO8910 cells 24 hr after treatment with Niclosamide. Actin was used as a loading control. The data expressed in right graphs represent the mean ± SD. **F.** Flow cytometry analysis of cell apoptosis after the ovarian carcinoma cells treated with different concentrations of Niclosamide for 24 hr. Cells were collected and stained with Annexin V-fluorescein isothiocyanate (FITC) and PI. Data are presented as mean ± SD.

Previous studies have identified the anti-cancer effects of Niclosamide in multiple cancer types and several signaling pathways, including Wnt/β-catenin, mTOR, STAT3, NF-κB, and Notch 18. In the present studies, Niclosamide showed tumor-suppressive activity in SKOV3 and HO8910 ovarian cancer cells as confirmed by a dose-dependent decrease in cell viability (**Figure 1A**). Similarly, cell growth and colony formation assays revealed Niclosamide significant reductions in cell and colony numbers, as well as morphological changes in response to Niclosamide (**Figure 1B-D**). Niclosamide examination of tumor growth associated pathways and MEK1/2-ERK1/2 signaling associated molecules revealed inactivation of MSK1, MEK1/2, and ERK1/2 as well as reduction of K-ras in Niclosamide-treated cells (**Figure 1E**). To further confirm the effect of ERK1/2 inhibition on cell growth, ERK1/2 specific inhibitor SCH772984 was used to treat SKOV3 and HO8910 cells, we found ERK1/2 was significantly inactivated and cell growth was decreased (**Figure S1A and B**). Niclosamide also initiated apoptosis in a pool of SKOV3 and HO8910 cells, suggesting a further mechanism to explain Niclosamide suppression of cancer cell growth (**Figure 1F**). These data confirmed that Niclosamide has promising tumor-suppressive activity in ovarian carcinoma cells.

### Niclosamide significantly interrupts mitochondrial respiration and dynamics

**Figure 2:**
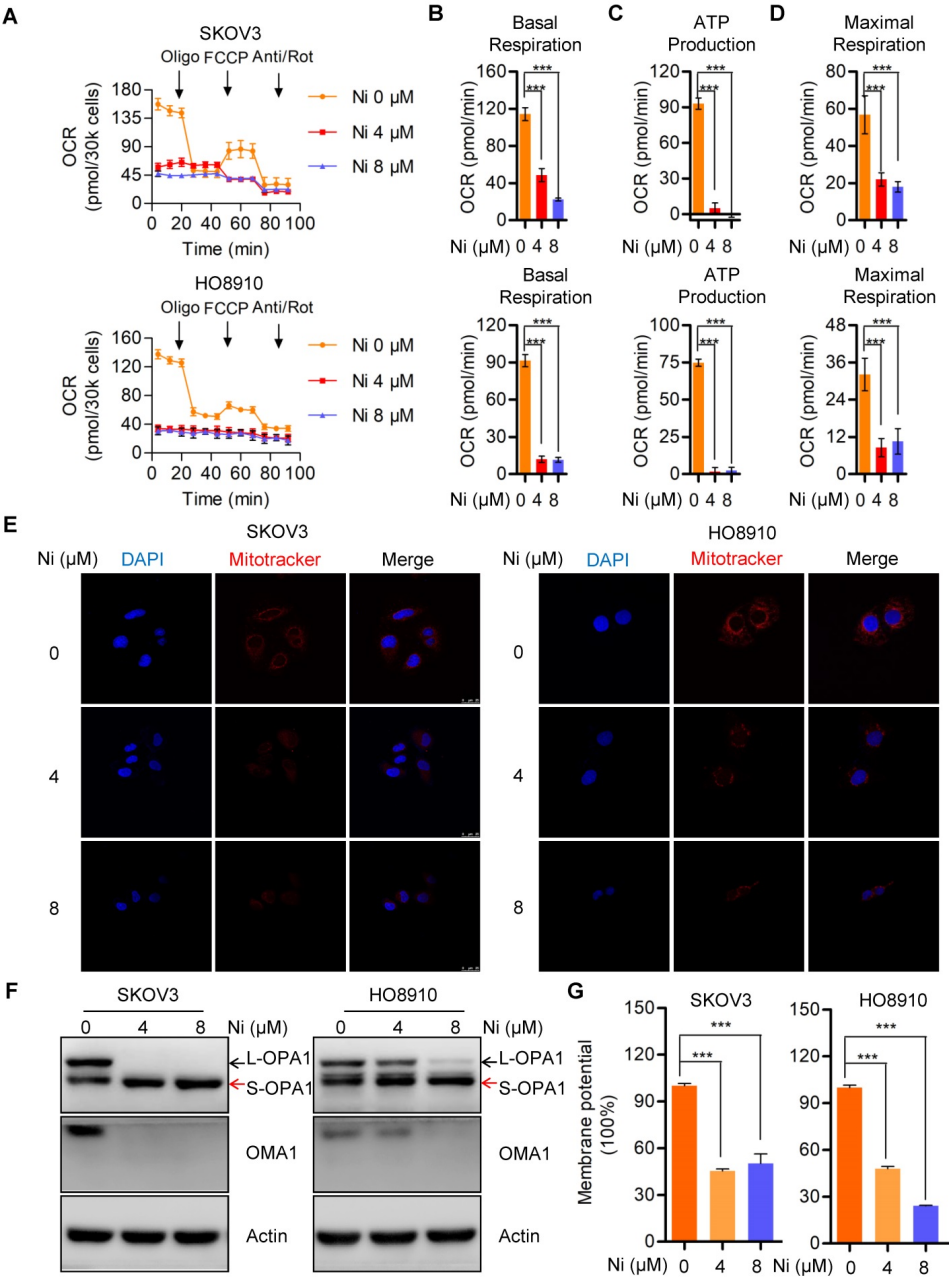
** Niclosamide significantly represses mitochondrial respiration and interrupt mitochondrial dynamics. A-D.** The intact cellular oxygen consumption rate (OCR) of Niclosamide treated SKOV3 and HO8910 cells under the indicated conditions were measured in real time using the Seahorse XF96 Extracellular Flux Analyzer. Basal OCR were measured at three time points, followed by sequential injections of the ATP synthase inhibitor oligomycin (1 μM), the uncoupler FCCP (1 μM), the complex I inhibitor rotenone (1 μM), and the complex III inhibitor antimycin A (1 μM). **E.** The mitochondrial morphology in Niclosamide treated SKOV3 and HO8910 cells were examined by DAPI and MitoTracker Red. **F.** Western blot analyses of OPA1 and OMA1 in Niclosamide treated SKOV3 and HO8910 cells. Actin was used as a loading control. **G.** SKOV3 and HO8910 cells were treated with or without Niclosamide (4, 8μM) for 24 hr. Cell samples were collected and stained with JC-1 MMP detection kit and analyzed by flow cytometry.

Mitochondria, as the major source of cellular ATP and other crucial metabolites, could represent a target for Niclosamide inhibition of ovarian carcinoma growth via effects on cell bioenergetics. The Seahorse bioenergetics analysis data confirmed that Niclosamide dramatically inhibited the overall mitochondrial oxygen consumption rate (OCR) (**Figure 2A**). Calculation of the OCR indexes of basal respiration, ATP production, and maximal respiration revealed dramatic reductions in all these indexes in Niclosamide-treated cells (**Figure 2B-D**). The mitochondria also showed morphological fragmentation changes and were fewer in number following Niclosamide addition (**Figure 2E**). OPA1, a crucial mitochondrial dynamics-associated protein, was cleaved in response to Niclosamide addition, in support of the observed mitochondrial fragmentation (**Figure 2F and Figure S2A**). To understand the cleavage of OPA1 in niclosamide treated cells, we further detected mitochondrial membrane potential (MMP) and found MMP was signidicantly decreased which indicated niclosamide caused mitochodnrial depolarization (**Figure 2G**).

### Aerobic glycolysis alteration due to Niclosamide administration

**Figure 3:**
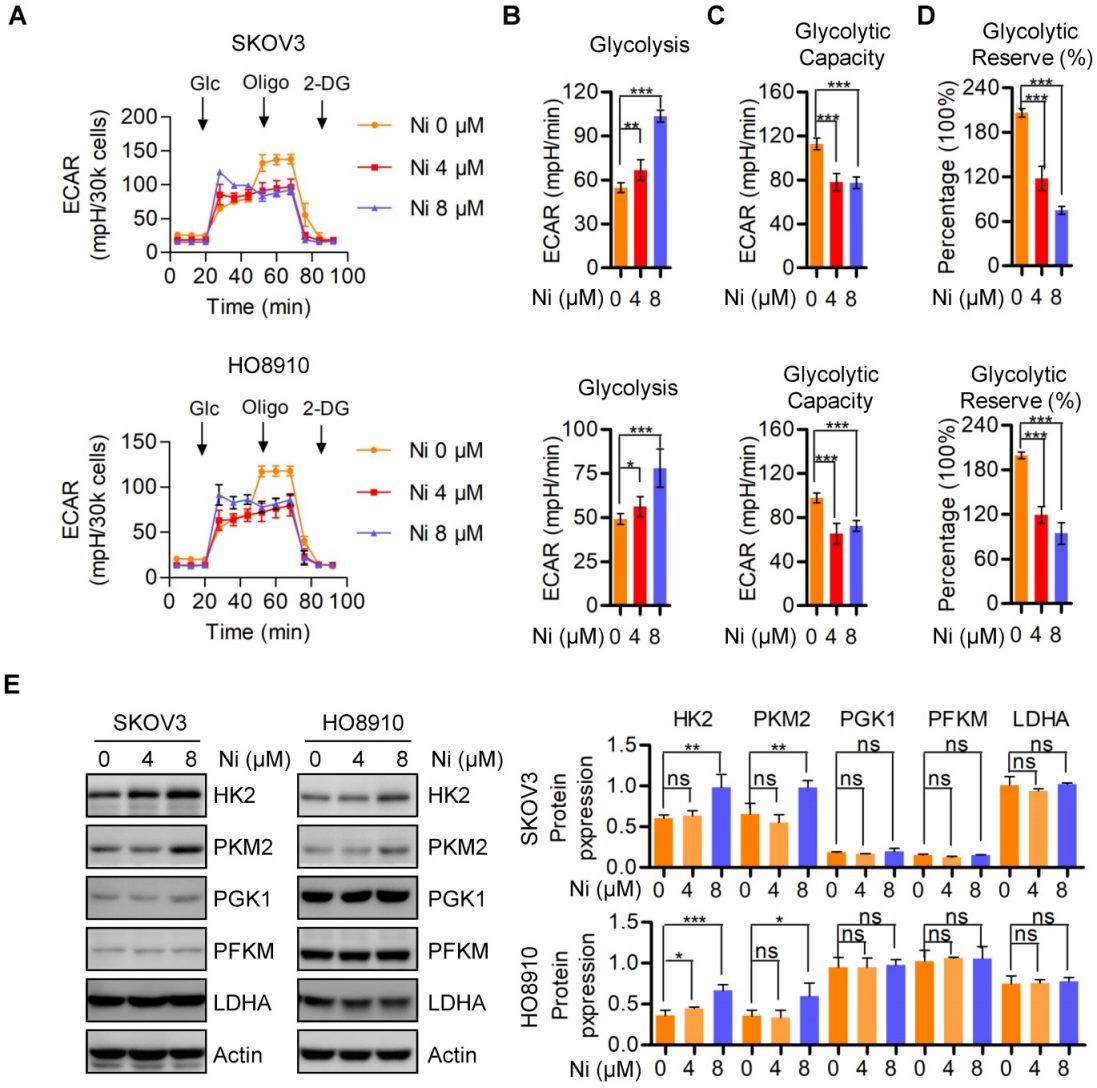
** Aerobic glycolysis alterations in response to nNiclosamide. A-D.** The extracellular acidification rate (ECAR) of Niclosamide treated SKOV3 and HO8910 cells under the indicated conditions were measured in real time using the Seahorse XF96 Extracellular Flux Analyzer. Basal ECAR were measured at three time points, followed by sequential injections of the Glucose (10 mM), Oligomycin (1 μM), and 2-DG (100 mM). Data are presented as mean ± SD. **E.** Western blot analysis of HK2, PKM2, PGK1, PFKM and LDHA in Niclosamide treated SKOV3 and HO8910 cells. Actin was used as a loading control.

In the 1920s, Dr. Otto Warburg found that cancer cells preferred to perform glycolysis even under normoxic conditions, and this phenomenon was named the “Warburg effect” 19. We evaluated mitochondrial respiration alterations due to Niclosamide by determining changes in aerobic glycolysis by measuring the extracellular acidification rate (ECAR) in vehicle and Niclosamide-treated SKOV3 and HO8910 ovarian carcinoma cells. The basal glycolysis was increased in the Niclosamide-treated cells, whereas the glycolytic capacity and glycolytic reverse were both decreased (**Figure 3A-D**). The expressions of glycolysis- associated enzymes HK2 and PKM2 were increased by Niclosamide treatment, but no significance change was found in PGK1, PFKM, and LDHA expression (**Figure 3C**).

### Niclosamide promotes ROS activated JNK-dependent cell apoptosis under glucose deprivation conditions

**Figure 4:**
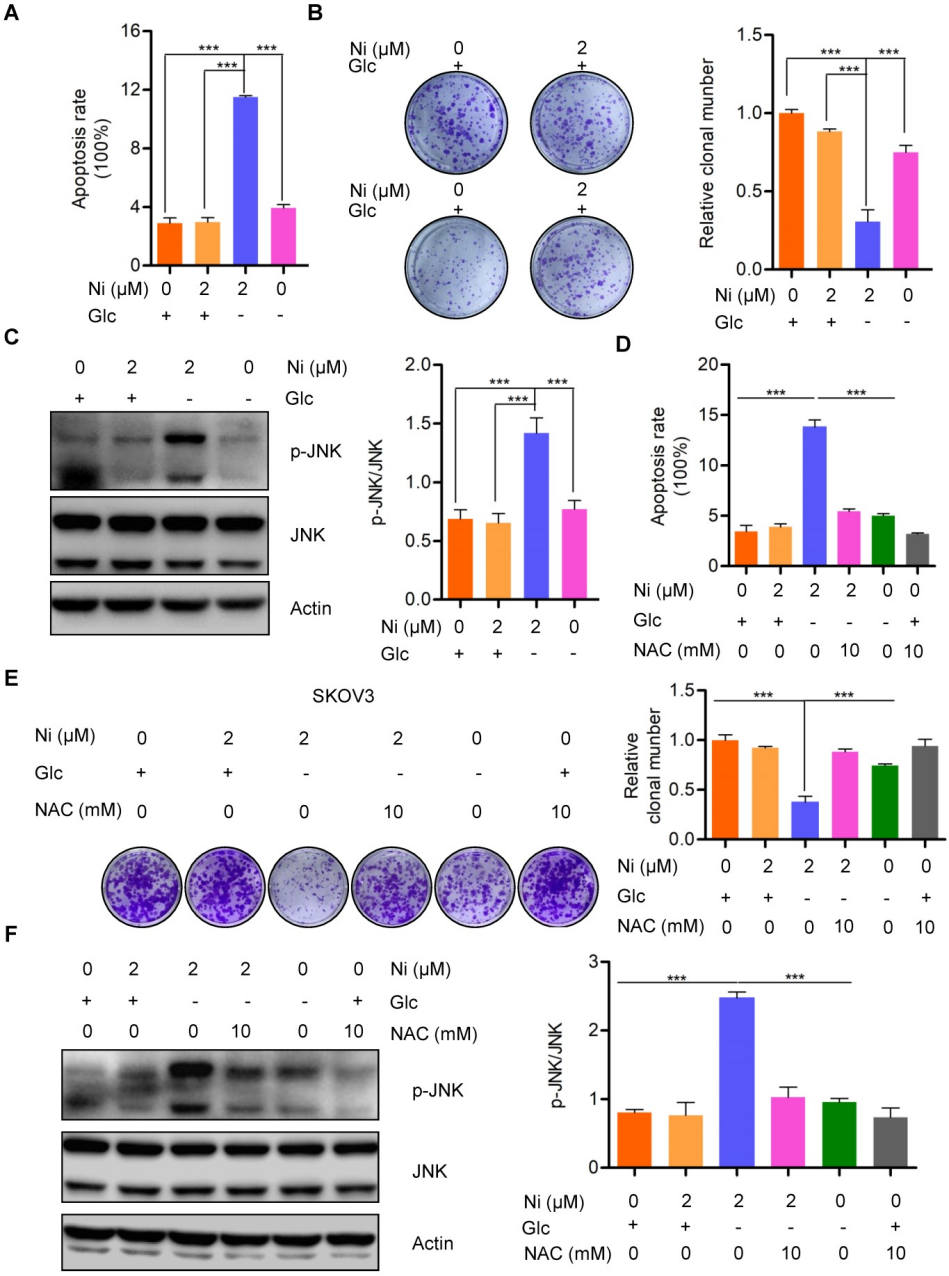
** Niclosamide promotes cell apoptosis effectivelly under the condition of glucose deprivation. A.** Flow cytometry analysis of cell apoptosis after the ovarian carcinoma cells treated with Niclosamide in the presence or absence of glucose for 12 hr. Cells were collected and stained with Annexin V-fluorescein isothiocyanate (FITC) and PI. Data are presented as mean ± SD. **B.** SKOV3 cells were treated with Niclosamide in the presence or absence of glucose for 12 hr, then subjected to culture for 3 days. Representative images of colonies and total colonies were recorded and calculated. Data are presented as mean ± SD.** C.** Western blot analysis of p-JNK and JNK in Niclosamide treated SKOV3 cells in the presence or absence of glucose for 12 hr. Actin was used as a loading control. Data are presented as mean ± SD.** D.** Flow cytometry analysis of cell apoptosis after the ovarian carcinoma cells were treated with Niclosamide and/or NAC in the presence or absence of glucose for 12 hr. Cells were collected and stained with Annexin V-fluorescein isothiocyanate (FITC) and PI. Data are presented as mean ± SD. **E.** SKOV3 cells were treated with Niclosamide and/or NAC in the presence or absence of glucose for 12 hr, and then cultured for 3 days. Representative images of colonies and total colonies were recorded and calculated. Data are presented as mean ± SD. **F.** Western blot analysis of p-JNK and JNK in Niclosamide treated SKOV3 cells with or without NAC and in the presence or absence of glucose for 12 hr. Actin was used as a loading control. Data are presented as mean ± SD.

The finding that Niclosamide decreased the basal cellular oxygen consumption rate while increasing the basal glycolytic rate indicated a stress response of metabolic reprogramming. The cellular bioenergetics data suggested that glucose deprivation could enhance the anti-tumor activity of Niclosamide. Niclosamide treatment (2 μM) of SKOV3 cells cultured under glucose deprivation conditions resulted in notable cell apoptosis when compared with cells cultured with glucose in the presence or absence of Niclosamide (2 μM) (**Figure 4A and B**). Consistently, we also found a dramatic suppression of cell growth in response to either Niclosamide treatment or glucose deprivation. Importantly, cells treated with Niclosamide under glucose deprivation showed the strongest tumor suppressive phenotype (**Figure S4A**). We also found marked activation of JNK, a crucial kinase belonging to MAPK family, in Niclosamide-treated cells under glucose deprivation, but not when cells were treated with Niclosamide alone or under glucose deprivation alone (**Figure 4C**). JNK is known to play dual roles in multiple carcinogenesis by activating tumor-associated signal pathways and by initiating cell apoptosis. Mechanistically, JNK activation occurs in a ROS-dependent manner, so we looked for a potential ROS involvement in cell apoptosis observed in response to Niclosamide treatment under glucose deprivation. We found that abolishment of the excess ROS production with NAC (10 mM) abrogated the Niclosamide-induced cell apoptosis under glucose deprivation (**Figure 4D**). Moreover, the strongest tumor suppressive phenotype was rescued by NAC (**Figure S4B**). We also found that the cell growth inhibition in Niclosamide-treated, glucose-deprived cells was also rescued by NAC (10 mM) administration (**Figure 4E**). Consistently, we found a significant inhibition of the increase in p-JN by NAC treatment of the Niclosamide-treated glucose- deprived cells, in agreement with the cell apoptosis findings (**Figure 4F**). These data suggested that Niclosamide enhanced cell apoptosis under glucose deprivation condition by a ROS-mediated activation of JNK.

### Niclosamide effectively suppresses *in vivo* ovarian carcinoma cell growth

**Figure 5:**
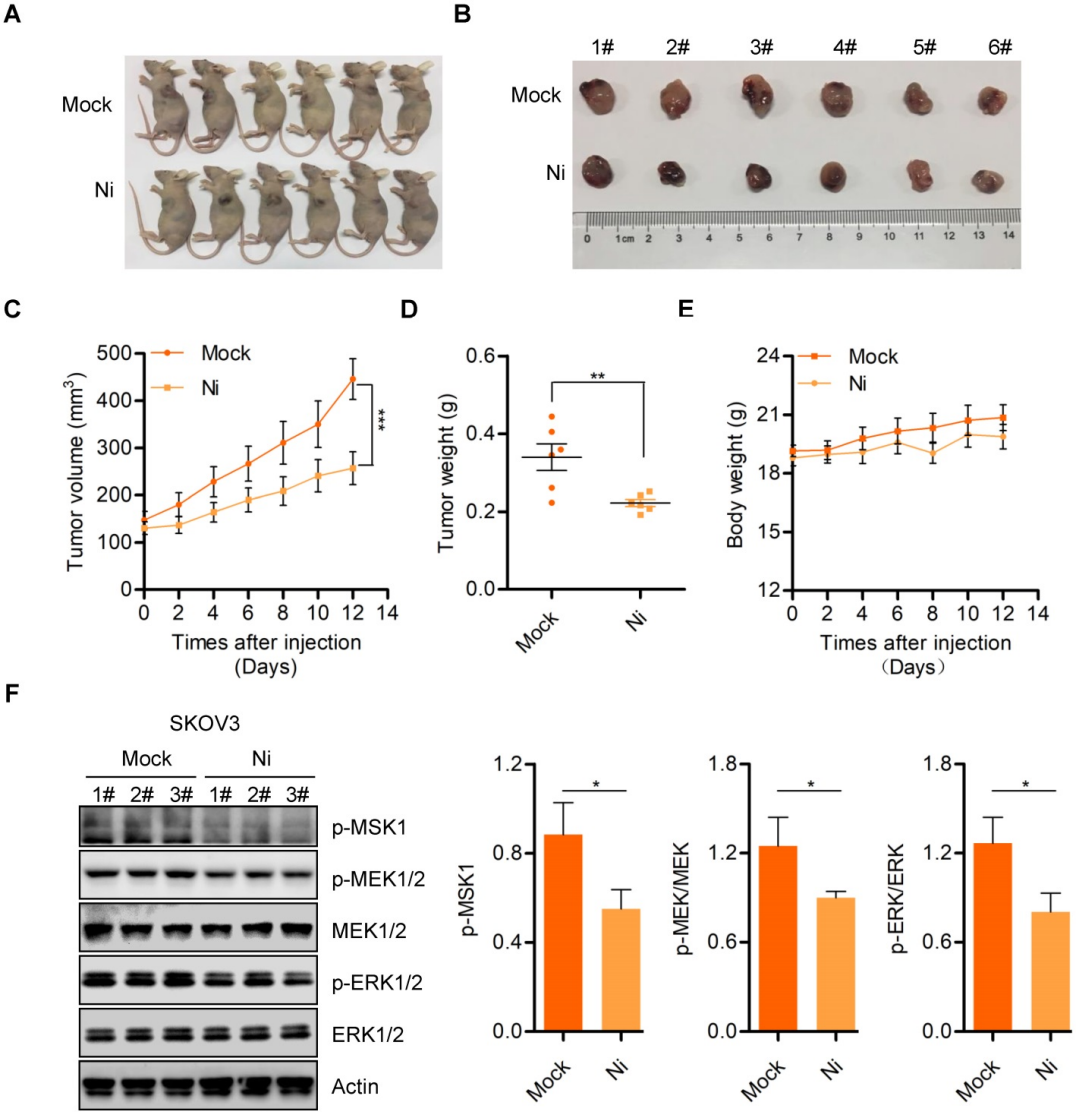
** Niclosamide inhibits ovarian carcinoma cell growth *in vivo*. A.** Representative images of sacrificed mice from physiological saline and Niclosamide treated groups. **B.** Representative images of dissected tumors obtained from mice after treatment with physiological saline and Niclosamide. **C.** Tumor volume in each mouse was monitored every two days. Data are presented as mean ± SE (n = 6). **D.** Changes in tumor weight in mice treated with saline or Niclosamide. Data are presented as mean ± SE (n = 6). **E.** Changes in body weight in saline- or Niclosamide-treated mice. Data are presented as mean ± SE (n = 6). **F.** Western blot analyses of p-MSK1, p-MEK1/2, MEK1/2, p-ERK1/2, and ERK1/2 pathways in tumor tissues derived from saline- or Niclosamide-treated mice. Actin was used as a loading control.

We further confirmed the anti-tumor activity of Niclosamide *in vivo* using a nude mouse transplanted- tumor model to evaluate the possible tumor- suppressive properties of Niclosamide. Consistent with the *in vitro* carcinoma cell data, Niclosamide significantly suppressed tumor growth in this nude mouse model (**Figure 5A and B**). The tumor volume and tumor weight of stripped tumor sections were notably decreased by Niclosamide administration (**Figure 5C and D**). Importantly, the nude mice showed no significant biological toxicity in response to Niclosamide treatment, supporting the potential for Niclosamide as an anti-cancer agent for ovarian carcinoma therapy (**Figure 5E**). We also explored potential mechanisms for the *in vivo* suppression of tumor growth by Niclosamide by western blot analysis of tumor sections. We found decreased expression of p-MSK1, p-MEK1/2, and p-ERK1/2 in response to Niclosamide treatment, in agreement with the *in vitro* data (**Figure 5F**). To further confirm the effect of Niclosamide on OPA1 cleavage *in vivo*, we analyzed OPA1 level in mock and Niclosamide administrated nude mice tumor sections and found OPA1 was cleaved which was consistent with the data *in vitro* (**Figure S5A**). These data further supported the anti-tumor efficacy of Niclosamide against ovarian carcinoma.

## Discussion

In the present study, we showed that Niclosamide is effective at suppression ovarian carcinoma cell growth both *in vitro* and *in vivo* and it inactivates MEK1/2-ERK1/2 signaling. Importantly, we demonstrated that cellular bioenergetics was dramatically repressed by Niclosamide addition. Interestingly, Niclosamide triggered ROS-mediated JNK activation to promote cell apoptosis under glucose deprivation, suggesting that Niclosamide may be a promising compound for ovarian cancer therapy.

Metabolic reprogramming is one of the important features of tumor cells. Cancer cell must meet new requirements for materials, energy, and redox forces to support their rapid proliferation, so tumor cells commonly show reprograming of metabolic pathways 20. Metabolic reprograming changes the levels or types of specific intra- and extra-cellular metabolites, and this action promotes tumor growth by modulating gene expression, cell status, and the tumor microenvironment. Glucose metabolism, glutamine metabolism, and lipid metabolism are the most significant metabolic pathways that commonly reprograming in tumor cells. Targeting metabolic reprograming can therefore significantly inhibit tumor growth and promote apoptosis. Following the breakthrough studies in the 1920s by Dr. Otto Warburg, who showed the metabolic features of cancer cells, cellular bioenergetics processes have been confirmed as crucial for carcinogenesis and cancer progression due to their integration with metabolic processes 19. Niclosamide, an anthelmintic used against tapeworm infections, uncouples the electron transport chain from ATP synthase, thereby restricting ATP production, the energy supply for metabolism. Disruption of this crucial metabolic pathway has been determined as an efficacious strategy for cancer therapy 21. In the present study, we found a significant reduction in the overall OCR in response to Niclosamide, consistent with its known biological activity. Importantly, we found that dynamin-related GTPase (OPA1), a mitochondrial dynamics-associated molecule, underwent cleavage from L-OPA1 to S-OPA1 in response to Niclosamide, indicating mitochondrial depolarization. OPA1 is essential for maintaining mitochondrial morphology by modulating the balance between mitochondrial fusion and mitochondrial fission 22. Upon mitochondrial depolarization, L-OPA1 is cleaved to S-OPA1 by OMA1 and the AAA+ protease YME1L. Specifically, L-OPA1, but not S-OPA1, is essential for mitochondrial fusion, whereas accumulation of S-OPA1 promotes mitochondrial division. In cultured mammalian cells, stress conditions activate OMA1, leading to cleavage of L-OPA1 to S-OPA1 and inhibition of mitochondrial fusion, followed by mitochondrial division 23. Our data showed a greater accumulation of S-OPA1 in response to Niclosamide, which indicated imbalanced mitochondrial dynamics. Previous studies has demonstrated that OPA1 could be cleaved by Yme1L and OMA1 or auto-cleaved, we found Niclosamide treatment decreased both OMA1 and Yme1L level which indicated that Nicolsamide caused mitochondrial depolarization and further activated auto-cleavage of OPA1 (**Figure S2B**). Indeed, we found Niclosamide significantly regulates mitochondrial dynamics through downregulating MFN1, DRP1 and FIS1 which indicated decreased mitofusion and fission (**Figure S2C**). Moreover, based on the Mito-tracker staining, we hypothesized that whether Niclosamide could regulate mitochondrial content, our data showed PGC-1α and PPAR-α were both decreased upon Niclosamide addition, however, no significant change of c-myc was found in Mock and Niclosamide treated cells (**Figure S2D**). Proteolytic cleavage of OPA1 stimulates mitochondrial inner membrane fusion and couples fusion to oxidative phosphorylation. Moreover, OPA1-dependent mitochondrial cristae remodeling pathway controls atrophic, apoptotic, and ischemic tissue damage which highlight the crucial role of OPA1 in regulating biological functions and cell metabolism. Further work is needed to elucidate the mechanism by which Niclosamide promotes OPA1 cleavage and mitochondrial fission.

We also found a slight increase in the basal glycolytic rate and expression of the glycolytic enzymes HK2 and PKM2, despite the overall decrease in glycolysis. Our interpretation is that the increase in basal ECAR maybe a stress response arising from the inhibition of mitochondrial respiration. Mechanistically, we found that Solute Carrier Family 1, Member 5 (SLC1A5) was increased in Niclosamide-treated SKOV3 and HO8910 cells. A previous study showed that c-Myc up-regulates SLC1A5 to promote amino acid metabolism, which subsequently activates mTORC1 signaling to favor hepatocarcinogenesis 24, 25. Consistently, we also found increases in p-mTOR (Ser2448) as well as in HIF1-α (Data are not shown). HIF1-α regulation requires mTOR, and HIF1-α is a key modulator of cellular glycolysis, acting by controlling the transcription of certain glycolytic enzymes. We speculated that Niclosamide treatment may interrupt mitochondrial homeostasis and then may up-regulate SLC1A5 to activate mTOR signaling. This would then increase HIF1-α expression and ultimately enhance glycolysis to overcome the shortage of ATP and other metabolites. However, we found no significant change of glucose transporter Glut1 upon Niclosamide addition which suggested Niclosamide caused increase of glycolysis was independent of glucose uptake (**Figure S3A**). Further work is needed to determine the detailed mechanism underlying this process.

We also investigated the effects of increased basal glycolysis on ovarian carcinoma cell survival. Due to a lack of vascularization, the center of a tumor is typically regarded as having a shortage of nutrients and oxygen, which suggests that Niclodamide might be more effective under a nutritional deficiency condition. Culture of ovarian carcinoma cells with or without glucose and subsequent treatment with Niclosamide, even at concentrations as low as 2 μM, revealed a significant promotion of apoptosis (almost 40%). Interestingly, the differences in apoptosis induced by treatment with Niclosamide (8 μM) alone or by glucose deprivation alone were less than 10%. This indicates that Niclosamide is more effective at inducing cell apoptosis under glucose restriction and suggests that a novel strategy for ovarian carcinoma therapy might be a combination of Niclosamide and a glycolysis inhibitor (such as 2-DG). Further study will confirm the efficacy of this combinatorial therapeutic strategy in ovarian carcinoma cells. Mechanistically, we found a significant activation of JNK in Niclosamide-treated cells subjected to glucose deprivation. JNK (c-Jun N-terminal kinase), also known as stress-activated protein kinase (SAPK), is a crucial member of the mitogen-activated protein kinase (MAPK) family in mammalian cells. The JNK signal transduction pathway is an important branch of the MAPK pathway that plays an important role in various physiological and pathological processes, such as the cell cycle, cell differentiation, reproduction, apoptosis, and cell stress 26. Recent studies have shown that ROS-dependent JNK activation is indispensable for celastrol-mediated autophagy and apoptosis 27. Our data using the ROS scavenger NAC also confirmed that Niclosamide-induced apoptosis under glucose deprivation is ROS-dependent. The role of other MAPK molecules (such as the p38 MAPK) in this process needs further confirmation.

Collectively, our data support Niclosamide as an effective suppressor of ovarian carcinoma growth through the disruption of cellular bioenergetics and interruption of mitochondrial dynamics. Importantly, we demonstrated greater Niclosamide-induced apoptosis under a glucose deprivation condition due to activation of ROS-dependent JNK signaling. These data suggest that Niclosamide may be a promising and potent drug for ovarian carcinoma therapy.

## Supplementary Material

Supplementary figures and tables.
