# Supplementary material for: Niclosamide inhibits ovarian carcinoma growth by interrupting cellular bioenergetics
Source: J Cancer. 2020 Mar 13;11(12):3454–66. doi: 10.7150/jca.41418 (PMC7150452; doi:10.7150/jca.41418)
Supplement: Supplementary file 1 — Supplementary figures and tables. [file jcav11p3454s1.pdf]

Niclosamide inhibits ovarian carcinoma growth by interrupting cellular bioenergetics

Fugen Shangguan[a,b]*, Yan Liu[c]*, Li Ma[c], Guiwu Qu[d], Qing Lv[d], Jing An[e], Shude Yang[f], Bin Lu[a#], Qizhi Cao[c, d#]

[a.] Protein Quality Control and Diseases Laboratory, Attardi Institute of Mitochondrial Biomedicine, School of Laboratory Medicine and Life Sciences, Wenzhou Medical University, Wenzhou, Zhejiang 325035, P.R. China

[b.] Key Laboratory of Diagnosis and Treatment of Severe Hepato-Pancreatic Diseases of Zhejiang Province, the First Affiliated Hospital of Wenzhou Medical University, Wenzhou, Zhejiang, 325000, China.

[c.] Department of Immunology, School of Basic Medical Sciences, Binzhou Medical University, Yantai, Shandong 264003, P.R. China

[d.] Anti-aging Research Institution, Binzhou Medical University, Yantai, Shandong 264003, P.R.China

[e.] School of Medicine, University of California - San Diego, La Jolla, CA 92037, USA

[f.] School of Agriculture, Ludong University, Yantai, Shandong 264025, P.R.China

*These authors contributed equally to this work.

[#]***Corresponding to*** **Prof. Qizhi Cao**, School of Basic Medical Sciences, Binzhou Medical University, No. 346, Guanhai Road, Yantai, Shandong 264003, China. Tel: +86-535-6913227 E-mail address: caoqizhi@126.com; **Prof. Bin Lu**, School of Laboratory Medicine and Life Sciences, Wenzhou Medical University, University-Town, Wenzhou, Zhejiang 325035, China; E-mail address: lubmito@wmu.edu.cn.

Supplemental Materials and methods

Cell lines and cell culture

The SKOV3, HO8910 human ovarian cancer cell lines were obtained from the Cell Bank of the Chinese Academy of Sciences (Shanghai, China). SKOV3 cells were cultured in RPMI-1640 medium (Life Technologies, Grand Island, NY) supplemented with 10% fetal bovine serum (FBS, Gemini, USA) and antibiotics (100 units/mL penicillin and 100 units/mL streptomycin) and HO8910 cells were cultured in DMEM medium supplemented with 10% fetal bovine serum and antibiotics. The cells were incubated at 37°C in a humidified incubator with 5% CO_2. When the cultures reach approximately 50-70% confluence, the cells were treated with various concentrations of drugs. Dimethyl sulfoxide (DMSO) was used as a vehicle control. All cell lines were mycoplasma free and authenticated by the Cell Bank of the Chinese Academy of Sciences.

Anti-proliferation assays

The effects of SCH772984 on the proliferation of SKOV3 and HO8910 cell lines were evaluated using Cell Counting Kit-8 (CCK-8). Briefly, SKOV3 andHO8910 cells were seeded into 96-well plates at a density of 3×10^3 cells/well and incubated overnight at 37°C with 5% CO_2. Cells were then treated with gradient concentrations (0, 2 μM) of Niclosamide for 1, 2, 3, and 4 days, and followed by incubating with Cell Counting Kit-8 (CCK-8) for 2 hr at 37°C, respectively. The absorbance at 450 nm was measured using a Varioskan Flash microplate reader (Thermo Scientific, Waltham, MA).

Western blot analysis

The ovarian cancer samples were washed three times with ice-cold PBS and homogenized using a homogenizer (Kinematica AG, Luzern, Switzerland) in 1.5 mL tissue RIPA lysis buffer (50 mM Tris-HCl, pH 7.4, 1.0% Triton X-100, 1% sodium deoxycholate, 0.1% SDS, and 150 mM NaCl) supplemented with protease inhibitor cocktail tablet, NaF (1 mM) and Na_3VO_4 (1 mM). Tissue homogenates were cleared by centrifugation at 13,000 rpm for 25 min at 4°C, and the supernatants were collected in clean microcentrifuge tubes on ice. A similar procedure was used to prepare whole cell extracts from cells. Briefly, SKOV3 and HO8910 cells were washed with ice-cold PBS and lysed in RIPA lysis buffer supplemented with protease and phosphatase inhibitors on ice for 20 min, followed by centrifugation at 13,000 rpm for 30 min at 4°C, and the supernatants were collected. Protein concentrations of the tissue homogenates or whole cell extracts were determined using the Pierce BCA protein assay kit. Tissue or cell extracts equivalent to 20 μg total protein were resolved in 10% SDS-PAGE gels followed by electrophoretic transfer onto PVDF membrane (0.22 μM, Bio-Rad, Hercules, CA) in Tris-glycine buffer. Blots were blocked at room temperature for 1.5 hr in 5% non-fat milk in Tris-buffered saline (TBS)-Tween (TBS-T) on a shaker, and then incubated with the primary antibodies in 5% non-fat milk TBS-T overnight at 4°C. The membrane was washed in TBS-T for at least 3 × 10 min and then incubated with horseradish peroxidase (HRP)-conjugated anti-rabbit or anti-mouse immunoglobulin G at room temperature for 1 hr with gentle shaking. Immunoreactive proteins were detected by ECL reagent according to the manufacturer’s protocol (Biyetime bioteconology).

FACS analysis for cell apoptosis

For apoptosis analysis, the SKOV3 and HO8910 cells were treated with gradient concentrations of Niclosamide (0, 4, and 8 μM) for 24 hr or with Niclosamide and/or NAC in the presence or absence of glucose for 12 hr; cells were then collected and incubated with Annexin V-FITC/ PI (BD, San Jose, CA) in the dark at room temperature for 20 min, according to the manufacturer's protocol. Thereafter, cell samples were analyzed immediately using a BD Accuri^{TM} C6 flow cytometer (BD, Franklin Lakes, NJ).

.

Figure S1

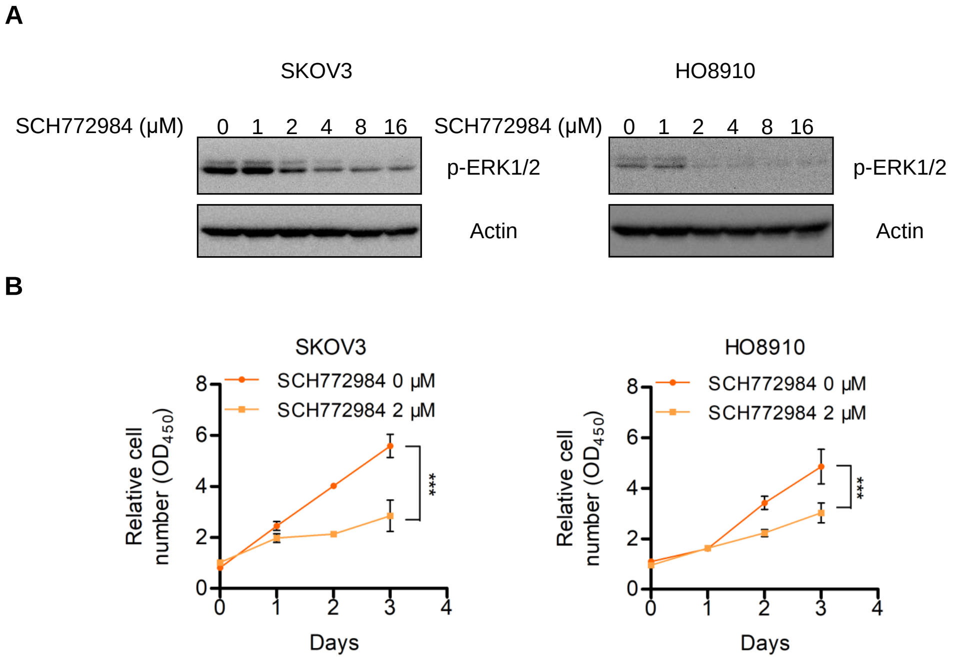

Figure S1. ERK inhibitor SCH772984 is effective to suppress ovarian carcinoma cell growth. A. Western blotting analyses of p-MEK1/2 and Actin in SKOV3 and HO8910 cells 48 hr after treatment with SCH772984. **B.** The cell viability was determined by CCK-8 Cell Proliferation and Cytotoxicity Assay Kit according to the manufacturer's instructions after treating with ERK inhibitor SCH772984.

Figure S2

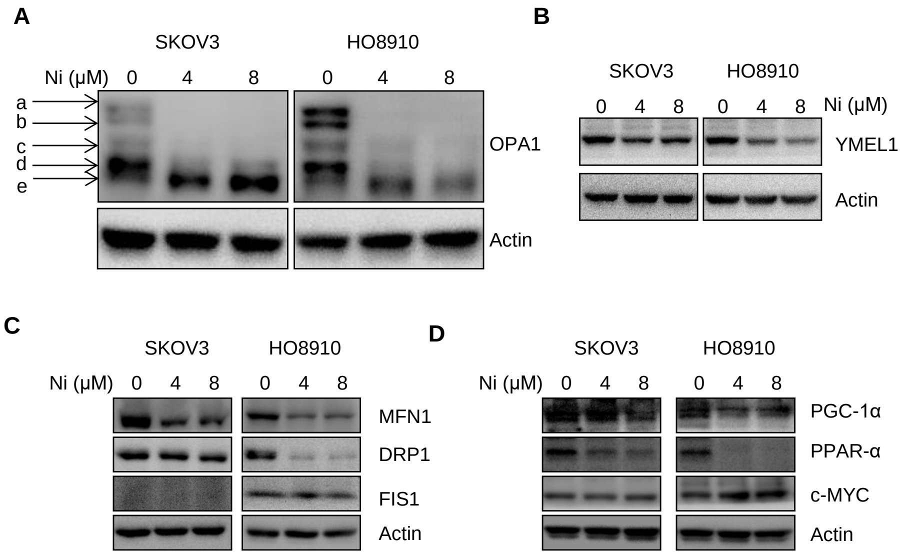

Figure S2. Niclosamide significantly represses mitochondrial respiration and interrupt mitochondrial dynamics. A. Western blot analyses of OPA1 and Actin in SKOV3 and HO8910 cells by 6% SDS-PAGE. **B.** Western blotting analyses of YmeL1 and Actin in SKOV3 and HO8910 cells. **C.** Western blotting analyses of MFN1, DRP1, FIS1 and Actin in SKOV3 and HO8910 cells. **D.** Western blot analyses of PGC-1α, PPAR-α, c-MYC and Actin in SKOV3 and HO8910 cells.

Figure S3

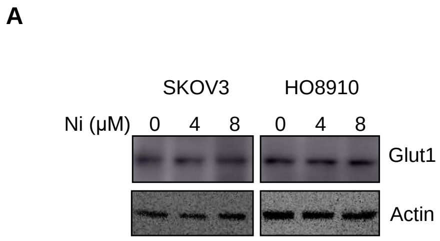

Figure S3. A. Western blotting analyses of GLUT1 and Actin in SKOV3 and HO8910 cells.

Figure S4

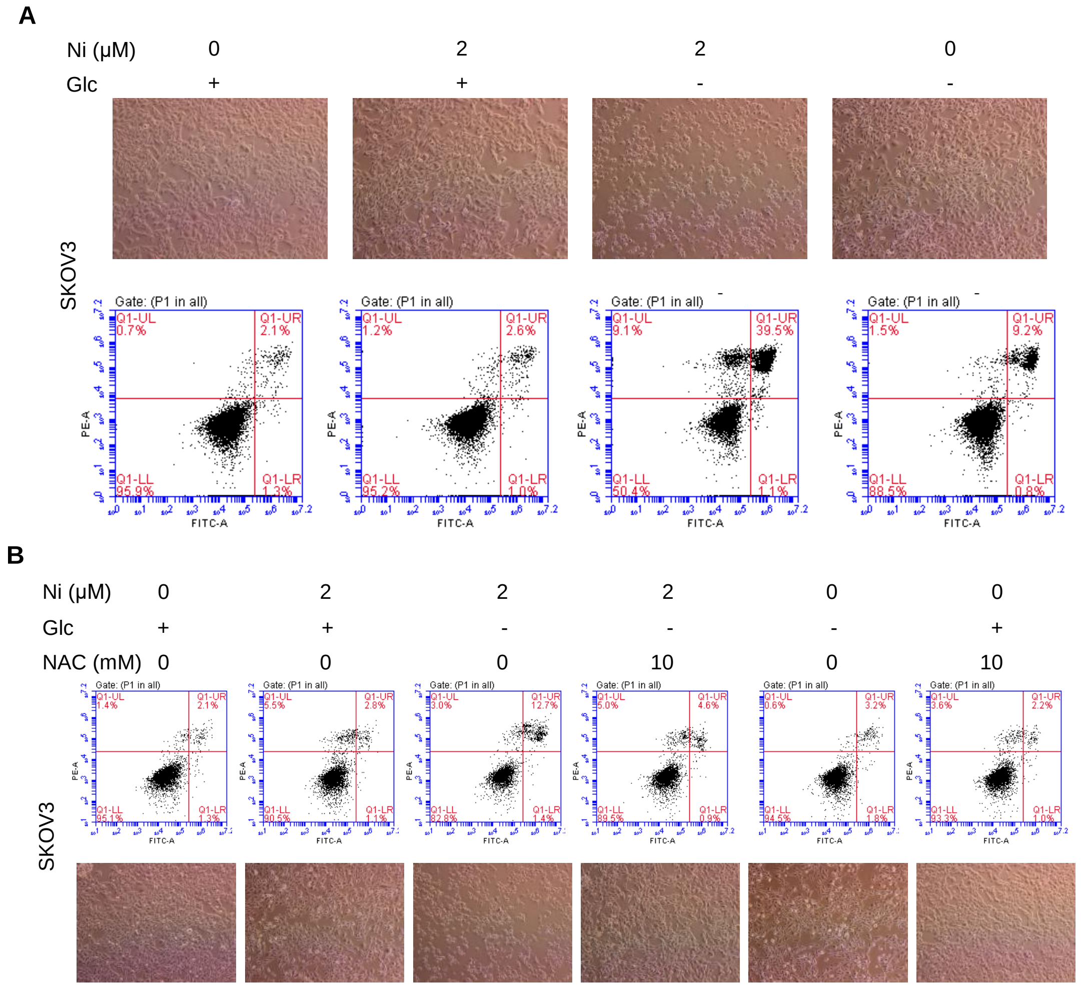

Figure S4. Niclosamide is more effective to promote cell apoptosis under glucose deprivation condition. A. Representative image of cellular morphology were taking after SKOV3 cells were treated with Niclosamide with or without glucose for 12 hr. Flow cytometry analysis of cell apoptosis after the ovarian cancer treated with Niclosamide with or without glucose for 12 hr. Cells were collected and stained with Annexin V-fluorescein is othiocyanate FITC and PI. **B.** Representative images of cellular morphology were taking after SKOV3 cells were treated with Niclosamide and/or NAC with or without glucose for 12 hr. Flow cytometry analysis of cell apoptosis after the ovarian cancer treated with Niclosamide and/or NAC with or without glucose for 12 hr. Cells were collected and stained with Annexin V-fluorescein is

othiocyanate FITC and PI.

Figure S5

A

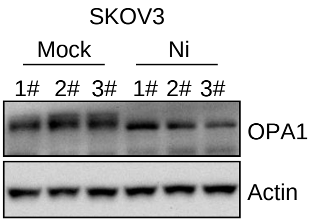

Figure S5. Niclosamide promotes OPA1 cleavage *in vivo*. A. Western blot analyses of OPA1 and Actin in tumor tissues derived from Mock- or Niclosamide-treated mice. Actin was used as a loading control.
